# Falsely high patient state index during cardiopulmonary bypass with intra-aortic balloon pumping: a case report

**DOI:** 10.1186/s40981-019-0299-x

**Published:** 2019-11-29

**Authors:** Shihoko Iwata, Michiyoshi Sanuki, Makoto Ozaki

**Affiliations:** 1 Department of Anesthesiology, Tokyo Women’s Medical University Hospital, 8-1 Kawada-cho, Shinjuku-ku, Tokyo, 162-8666 Japan; 2 Department of Anesthesiology, Critical Care and Pain Medicine, National Hospital Organization Kure Medical Center and Chugoku Cancer Center, 3-1 Aoyama-cho, Kure, Hiroshima, 737-0023 Japan

**Keywords:** Electroencephalography, Patient state index, Cardiopulmonary bypass, Intra-aortic balloon pumping, Diagnostic error, Artifact

## Abstract

**Background:**

The patient state index (PSI) is a parameter of a four-channel electroencephalography (EEG)-derived variable used to assess the depth of anesthesia. A PSI value of 25–50 indicates adequate state of hypnosis, and a value of 100 indicates a fully awake state. Due to reduced interference from electronic devices like electrocautery, falsely high intraoperative PSI values are rarely reported. However, this case report cautions about falsely high PSI during cardiopulmonary bypass (CPB) with intra-aortic balloon pumping (IABP).

**Case presentation:**

A 68-year-old man was scheduled for coronary artery bypass graft surgery with IABP. General anesthesia was maintained using sevoflurane. Initial PSI was between 30 and 50 before CPB. Propofol was administered during CPB, and IABP provided pulsatile flow. IABP was stopped soon after the initiation of CPB, and the ascending aorta was partially clamped to anastomose the saphenous vein graft to the ascending aorta. The PSI value decreased drastically, but with resumption of IABP, the value increased to approximately 80, despite increasing the dose of anesthetics. Meanwhile, the EEG waveform was nearly flat. After discontinuing CPB, the PSI value returned to being extremely low. There was no evidence of intraoperative awareness or instrument trouble.

After reviewing the anesthesia record, the high PSI value was almost consistent with ongoing IABP during CPB. We suspect that the oscillation noise created by IABP during CPB erroneously influences the PSI algorithm, resulting in a falsely high PSI.

**Conclusions:**

Anesthesiologists should note that adherence to pEEG-derived values without discretion may cause errors when monitoring the depth of anesthesia.

## Background

Electroencephalography (EEG) waveforms and processed EEG (pEEG) are useful to prevent intraoperative awareness. Although bispectral index is the most widely used pEEG, it is falsely increased by electromyographic activity and electric device interference [1–5]. The patient state index (PSI) is a recently introduced parameter calculated based on a four-channel EEG by the SedLine® monitor (Masimo Co., Irvine, CA, USA) to assess the level of consciousness. Unlike bispectral index, PSI is hardly affected by electronic devices such as the electrocautery unit and thus provides reliable measures of perioperative consciousness levels [6–8] and there have been no reports of falsely increased PSI values perioperatively due to any machine-related oscillation noises. We report the case of a patient who underwent cardiopulmonary bypass (CPB) with intra-aortic balloon pumping (IABP) and showed an unexpectedly high PSI value despite administration of sufficient anesthetic agents.

## Case presentation

A 68-year-old man was scheduled for elective coronary artery bypass graft surgery. He had a past history of hypertension, bronchial asthma, cerebral infarction, and myelodysplastic syndrome and has been on hemodialysis because of autosomal dominant polycystic kidney disease. He was alert and preoperative laboratory data were unremarkable except white blood cell count 2.24 × 10^3^/μL, red blood cell count 3.83 × 10^3^/μL, platelet count 9.1× 10^4^/μL, creatinine 4.15 mg/dL, and B-type natriuretic peptide 161.9 pg/mL. Echocardiography demonstrated left ventricular ejection fraction 34%, mild aortic regurgitation, mild aortic stenosis, and mild mitral regurgitation. An IABP catheter (TRANS-RAY®, 7.5 Fr, 34 mL, Getinge AB, Lindholmspiren, Göteborg, Sweden) was inserted via the right femoral artery and connected to the console (Cardiosave® IABP Hybrid, Getinge AB), and circulatory support was started before surgery.

In the operating room, a radial arterial catheter was inserted and an electrode for monitoring PSI was placed on the forehead in addition to routine monitors. General anesthesia was induced with midazolam 3 mg and remifentanil 0.3 μg/kg/min and was maintained using sevoflurane in an air–oxygen mix after tracheal intubation. PSI value prior to CPB was between 30 and 50. Propofol was continuously infused with a target concentration of 3 μg/mL using an infusion pump (TERUMO TE-371, Diprifuser™, TERUMO, Tokyo, Japan), and heart rate was approximately 60 bpm during CPB. IABP was continuously operated using electrocardiogram-triggered mode (1:1) in order to provide pulsatile flow.

**Fig. 1:**
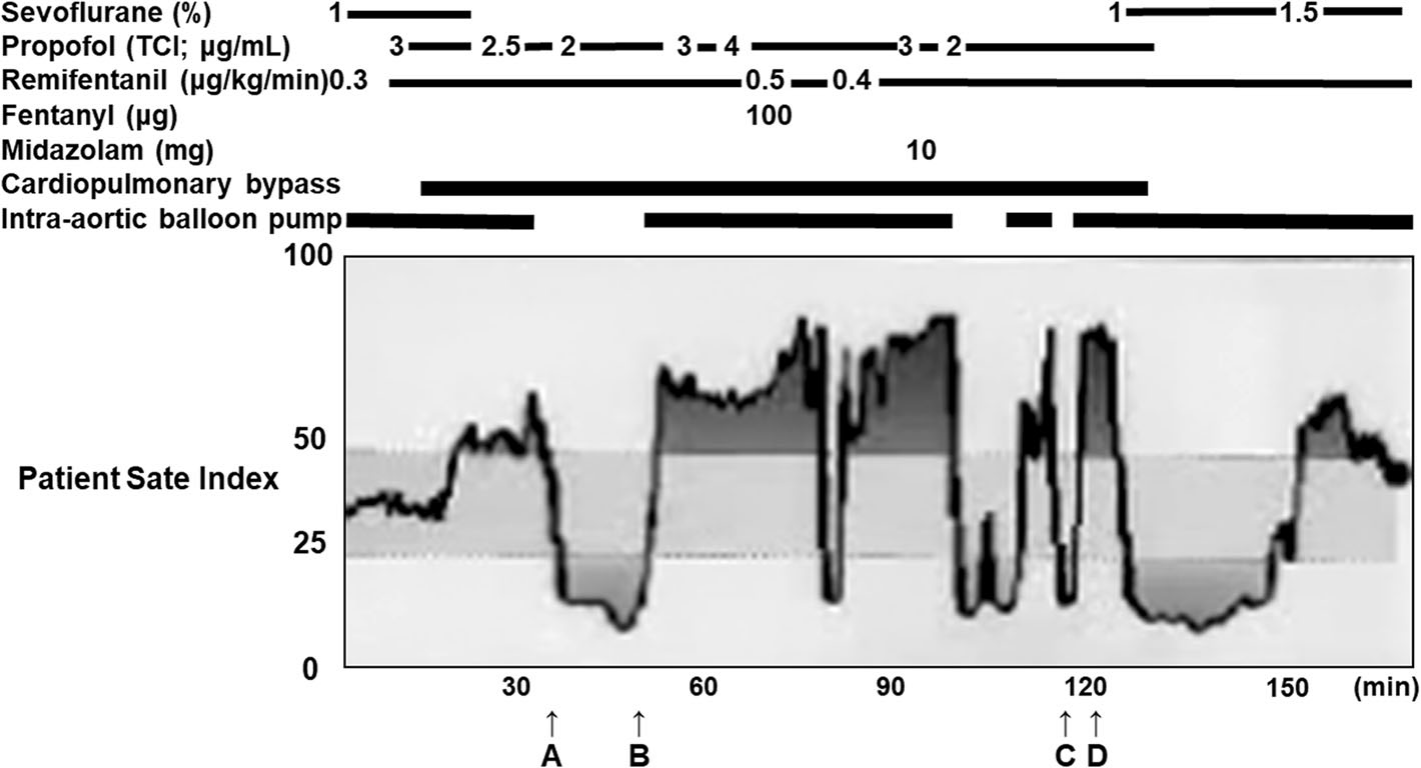
Patient state index from ascending aorta cannulation to after cardiopulmonary bypass. Patient state index was remarkably decreased after stopping intra-aortic balloon pumping (IABP) before partial clamping of the ascending aorta for anastomosis with the saphenous vein (**a**), rapidly increased after de-clamping the ascending aorta and restarting IABP (**b**), decreased after stopping IABP for hemostasis around the anastomosis (**c**), and again rapidly increased corresponding to the restart of IABP (**d**). Time 0 corresponds to ascending aortic cannulation

**Fig. 2:**
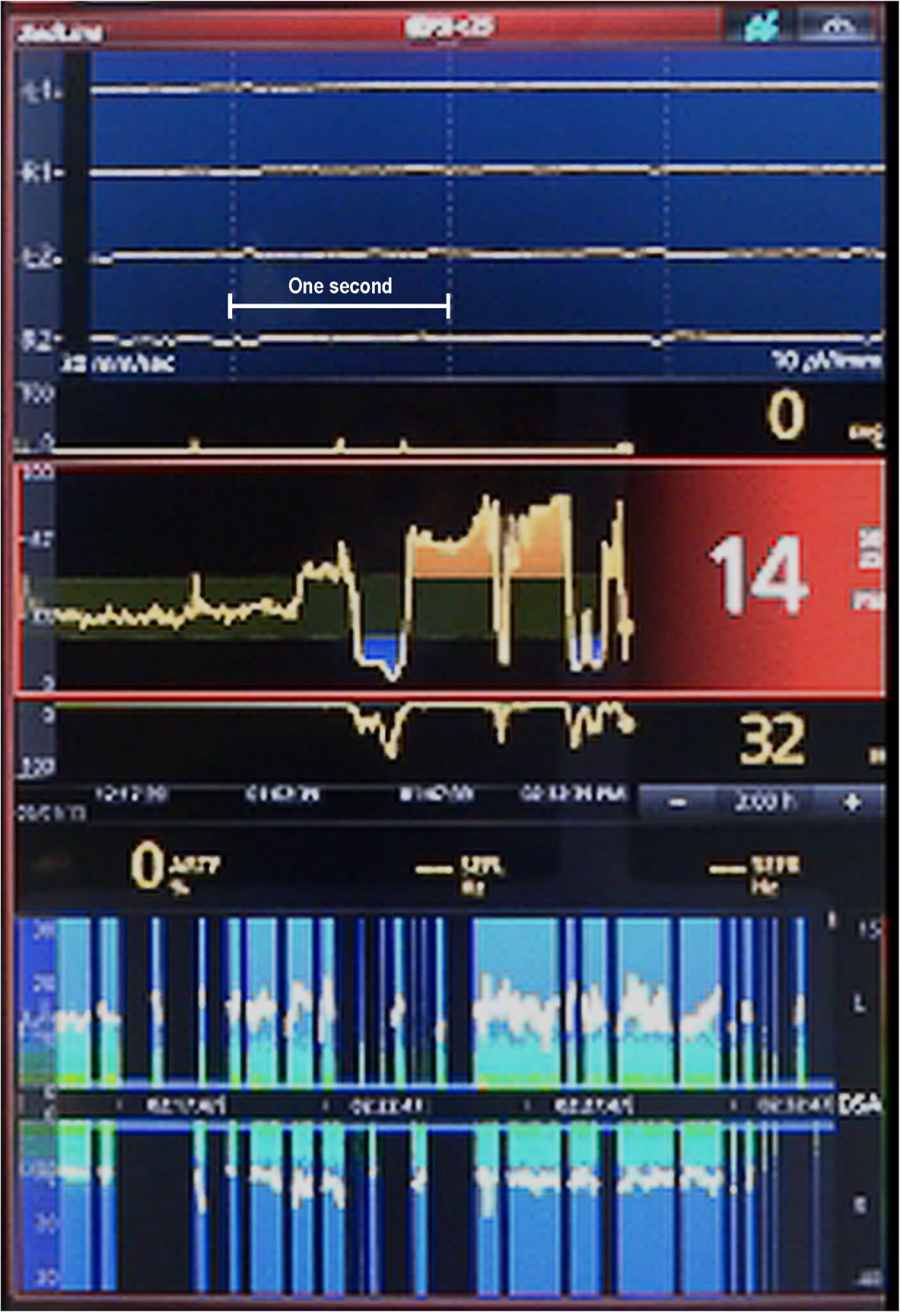
The display window of SedLine® without intra-aortic balloon pumping. Electroencephalogram (EEG) (top), patient state index (PSI) and suppression ratio (middle), and color density spectral array (DSA) (bottom) are shown. PSI was 14 and suppression ratio was 32 with almost flat EEG at the time point C in Fig. 1. The vertical white dotted lines indicate each second, and a horizontal white line shows 1 s in EEG. DSA indicates time (*x*-axis), frequency (*y*-axis), and power (color scale) with 95% spectral edge frequency (white line). Vertical black lines with a blue bar at 0 Hz timeline in DSA, conveying periods of suppression, were observed when PSI was extremely low

**Fig. 3:**
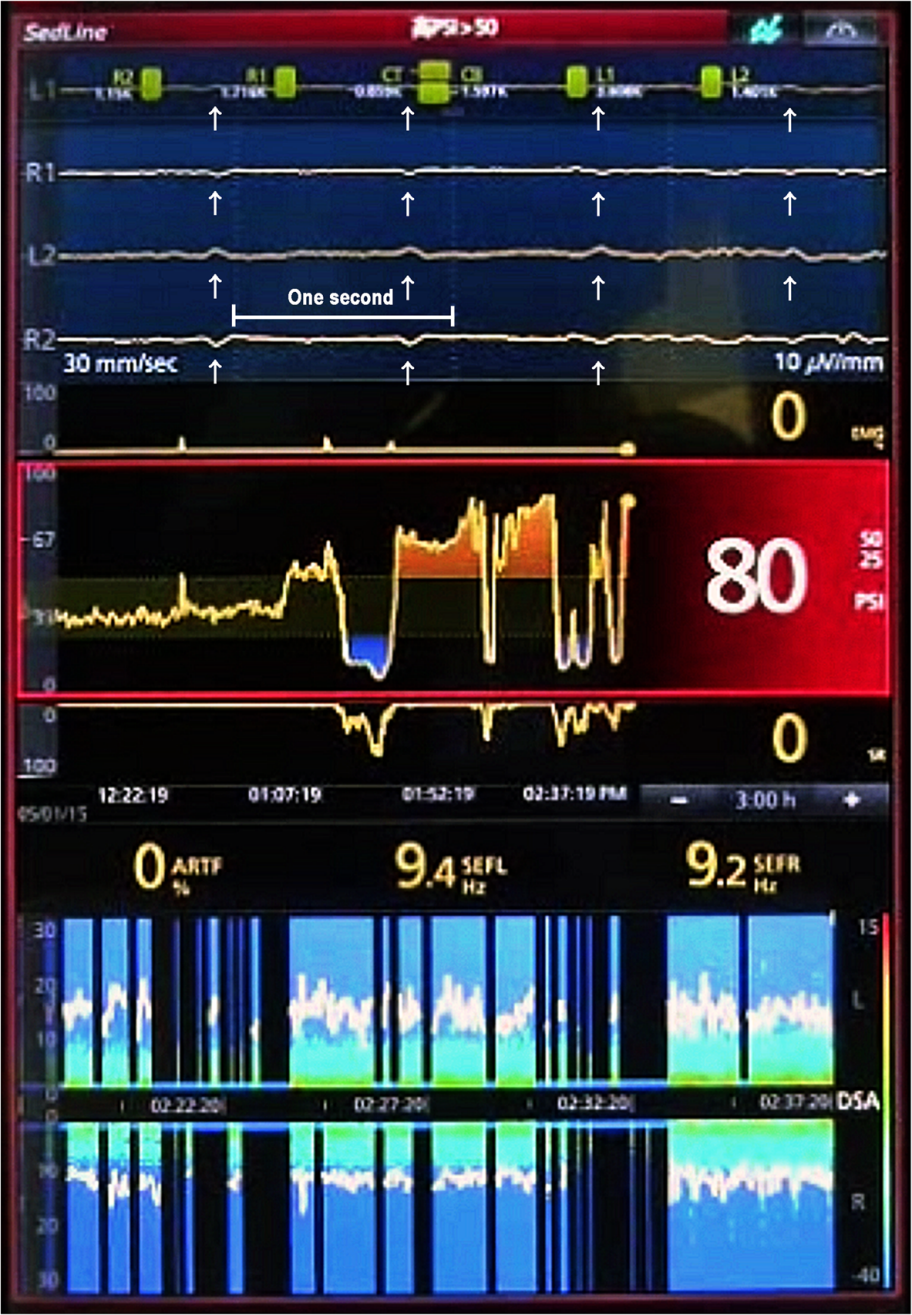
The display window of SedLine® after restarting intra-aortic balloon pumping. Patient state index (PSI) was increased from 14 to 80 after restarting intra-aortic balloon pumping (IABP) before weaning from cardiopulmonary bypass. Regular oscillation noises at about 60 Hz corresponding to the frequency of IABP (white arrow) are observed on almost flat EEG (top) at the time point D in Fig. 1. The vertical white dotted lines indicate each second, and a horizontal white line shows 1 s in EEG. Ninety-five percent spectral edge frequency was 9.4 Hz and 9.2 Hz on the left and right side, respectively. The density spectral array trend indicates the predominance of low frequency waves

PSI value was increased from 30 to 50 immediately after starting CPB and remarkably decreased after stopping IABP before partial clamping of the ascending aorta for anastomosis with the saphenous vein (Fig. 1). PSI was increased to approximately 70 abruptly after resuming IABP, which did not respond to an increase of infusion rate of propofol and remifentanil and to a bolus administration of fentanyl 100 μg or midazolam 10 mg. PSI values varied between 6 and 88 in accordance with the stopping and restarting of IABP. It decreased to 14 after stopping IABP for hemostasis around the anastomosis (Fig. 2) and again rapidly increased to 80 corresponding to restart of IABP before weaning from CPB (Fig. 3). EEG remained almost electrically silent, mean arterial blood pressure was around 40 mmHg, and rectal temperature was 35.3 °C during CPB with a flow rate of 2.5 L/min. Based on the EEG findings, we were certain that the patient was adequately anesthetized. After weaning the patient from CPB, the PSI value and EEG pattern spontaneously showed sedative status. Postoperatively, there was no evidence of intraoperative awareness, anesthetic drug delivery, or instrument trouble.

## Discussion

PSI is used to evaluate depth of anesthesia as well as to titrate the dose of anesthetics during the maintenance period. Although PSI is less affected by the electrocautery unit during surgery than bispectral index [6], safely monitoring a patient by blind obedience to a processed EEG values is not possible [9].

In our case, PSI showed unreasonable values during CPB. Reviewing the anesthesia record, continuous vital signs, and SedLine® (algorithm version: V1203) data, we noted that the PSI value fluctuated with the start and stop of IABP during CPB. The PSI value dropped to an extremely low level, and suppression ratio was high when IABP was stopped. Moreover, flat EEG, as shown by vertical black lines with a blue bar at 0 Hz in the density spectral array, was observed during this period (Fig. 2). On the other hand, the PSI value sharply increased despite almost unchanged EEG and spectral density, expect for small blips when IABP was started again. Of importance, SedLine® algorithm did not recognize the small blips as artifacts and showed “ARTF 0%” (Fig. 3).

We suspected that the oscillation noises existed during the entire anesthesia period. However, the algorithm of PSI confused artifacts, such as small blips as seen in Fig. 3 with fast waves of EEG because baseline EEG was suppressed to almost flat during CPB. On the other hand, PSI values would have been correctly calculated before and after CPB, because amplitude of the oscillation noise of IABP was too small compared with that of slow waves commonly observed during anesthesia. However, the reason why such small blips in almost flat EEG resulted in a PSI reading of 80 (Fig. 3) remains unclear because the details of PSI algorithm have not been completely clarified.

We found that 95% spectral edge frequency was fewer than 10 Hz and less than 13.5 Hz, indicating the threshold to differentiate intraoperative and pre-arousal state [10]. The density spectral array trend also indicates the predominance of slow EEG waves, suggesting that the patient was in the deep anesthesia state.

These findings showed that there is a possibility of misinterpreting the depth of anesthesia from PSI values alone, which could have dire consequences in overdosing general anesthetics, if anesthesiologists cling to pEEG-derived values without discretion or critical thinking. On the other hand, other parameters of such raw EEG waveforms, SEF, and DSA demonstrated that the patient was in a state of deep sedation when IABP worked during CPB despite falsely high PSI values.

The anesthesiologist should understand the changes in raw EEG expected during general anesthesia as well as the sources of non-EEG artifact and the unusual EEG waveforms that might occur. Additionally, for evaluating the depth of anesthesia properly, it is important to judge comprehensively, using the parameters of brain function monitoring, not only PSI values but also the waveforms of EEG, SEF, and DSA.

## Data Availability

Not applicable.

## Abbreviations

CPB: Cardiopulmonary bypass
DSA: Density spectral array
EEG: Electroencephalography
IABP: Intra-aortic balloon pumping
pEEG: Processed electroencephalography
PSI: Patient state index
SEF: Spectral edge frequency
